# Gallstones in a Looking-Glass: A Case Report on the Successful Laparoscopic Management of Cholelithiasis in Situs Inversus Totalis

**DOI:** 10.7759/cureus.67734

**Published:** 2024-08-25

**Authors:** Hafeez S Abdullah, Taif H Alomar, Ranad S Alamri, Abdullah A Alalawi

**Affiliations:** 1 General Surgery, King Fahad General Hospital, Medina, SAU; 2 College of Medicine, Taibah University, Madinah, SAU; 3 General Surgery, King Fahad General Hospital, Madinah, SAU

**Keywords:** surgery, hepatobiliary, difficult cholecystectomy, situs inversus totalis, cholelithiasis

## Abstract

Situs inversus is an uncommon congenital condition where the internal organs are arranged in a mirrored or reversed orientation within the body. In this unique anatomical variation, the placement of visceral organs is flipped, presenting a mirror-image configuration relative to their standard positions. While situs inversus itself does not predispose an individual to gallbladder disorders, the anatomical variation poses unique challenges for healthcare professionals in managing abdominal pathologies.

This case report describes the successful management of a 52-year-old male patient with situs inversus totalis who presented with gallstone-induced obstructive jaundice and underwent endoscopic retrograde cholangiopancreatography (ERCP) with stenting, followed by a laparoscopic cholecystectomy. The surgical procedure required exceptional visual-motor skills and extensive reorientation to accurately identify and navigate the left upper quadrant anatomy, which is the mirror image of the typical surgical approach. The case highlights the importance of thorough preoperative planning, comprehensive anatomical knowledge, and a multidisciplinary team approach to ensure favorable outcomes for patients with this rare condition.

## Introduction

Situs inversus is a fairly rare congenital disease characterized by a complete or partial mirroring of the internal organ placement within the body. In this anatomical anomaly, the visceral organs are positioned in a reverse or mirror-image orientation compared to their typical location [1]. The condition can manifest as either partial situs inversus, where only the thoracic or abdominal organs are affected, or total situs inversus, where the entire set of internal organs is reversed. Notably, situs inversus does not inherently predispose an individual to the development of gallbladder disorders. However, this anatomical variation requires heightened clinical suspicion and awareness among healthcare providers to avoid potential diagnostic confusion when evaluating patients with abdominal complaints [2].

The gallbladder typically resides attached to the right liver's lobe, but occasionally it can be found in aberrant locations, like the left side of the abdomen, the midline of the abdomen, or other unusual places. In patients with situs inversus totalis, where the complete set of internal organs is reversed, the gallbladder and liver are situated in the left upper abdominal quadrant, rather than the typical right upper quadrant location [3,4]. This anatomical variation poses unique challenges for healthcare professionals in examination and diagnosing, as it requires a complete reorientation of their understanding of the patient's internal organ placement and surgical landmarks. Several case reports have been documented in the medical literature describing the management of gallbladder pathologies in patients with situs inversus totalis [3].

Performing laparoscopic cholecystectomy, a minimally invasive surgical procedure to remove the gallbladder, in these individuals is clinically demanding. It requires exceptional visual-motor skills and extensive reorientation to accurately identify and navigate the left upper quadrant anatomy, which is the mirror image of the typical surgical approach [5]. The standard laparoscopic cholecystectomy usually requires the insertion of four trocar ports. Two 10 mm ports are placed near the umbilical area and blew the xiphoid process, while the other two 5 mm ports are positioned along the right midclavicular line and the anterior axillary line [1].

## Case presentation

A 52-year-old male patient presented to the outpatient clinic with a history of intermittent left upper quadrant abdominal pain for two-three months. The pain was colicky in nature, moderate in severity, and aggravated by eating fatty foods. The pain was partially relieved by regular analgesics. The patient also reported mild nausea and vomiting several times after eating, without specific characterization. The patient decided to seek medical help after developing jaundice and visited another healthcare facility, where he underwent proper evaluation and assessment. He was discovered to have situs inversus totalis, and endoscopic retrograde cholangiopancreatography (ERCP) with stenting was performed due to obstructive jaundice secondary to gallstones.

**Figure 1:**
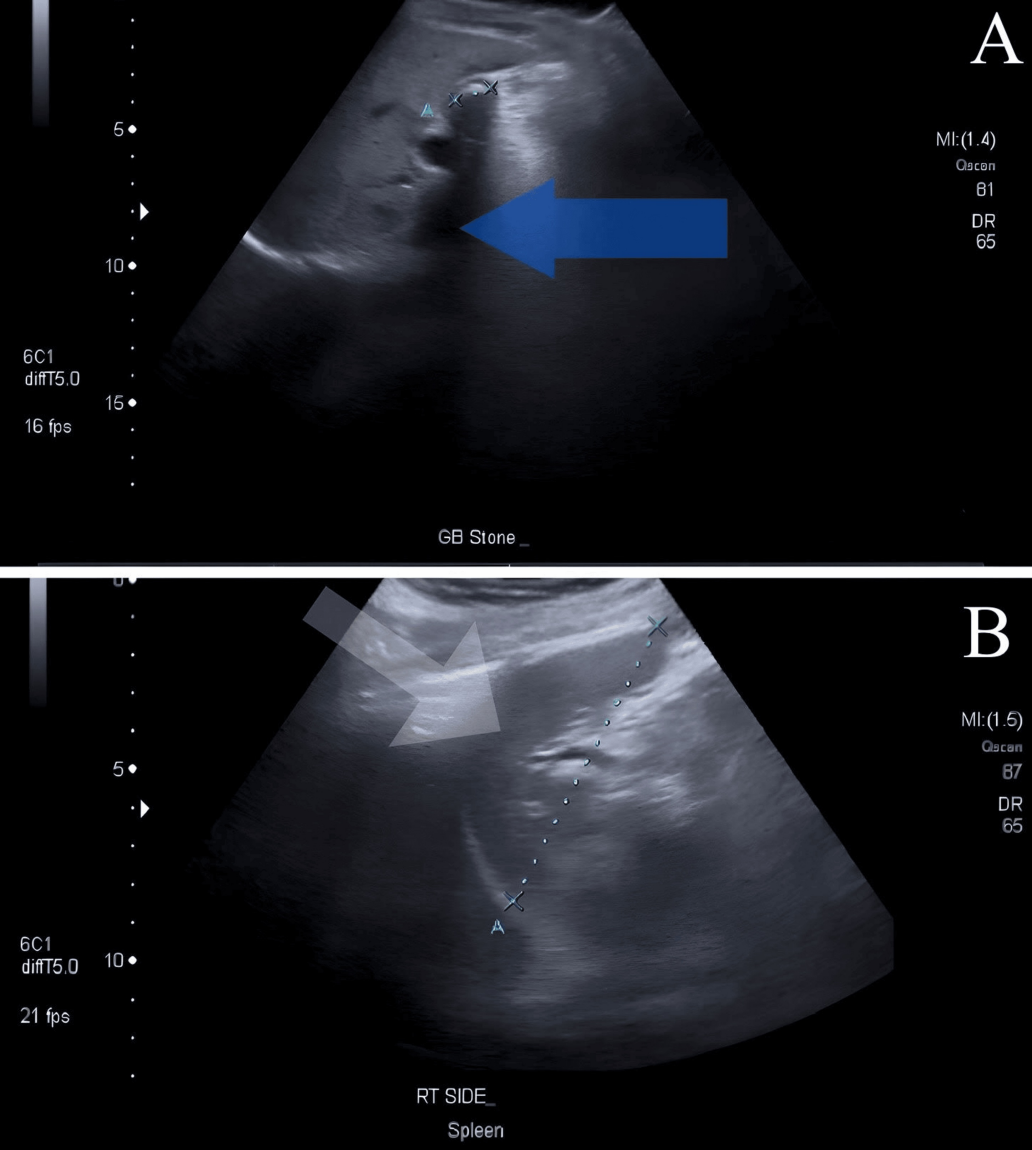
Abdomen US Multiple gallbladder stones with stone impacted in the neck (A) and posterior shadow (blue arrow). The spleen (white arrow) shows homogeneous and uniform parenchymal echogenicity and it is positioned on the right side (B). US: Ultrasound

**Figure 2:**
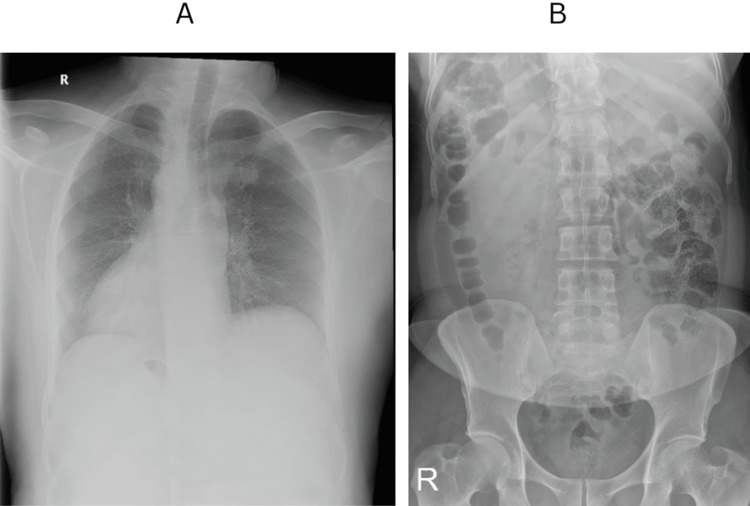
Posterior-anterior X-rays Posterior-anterior chest X-ray (A) and abdominal X-ray (B) showing dextrocardia and transposition of organs.

**Figure 3:**
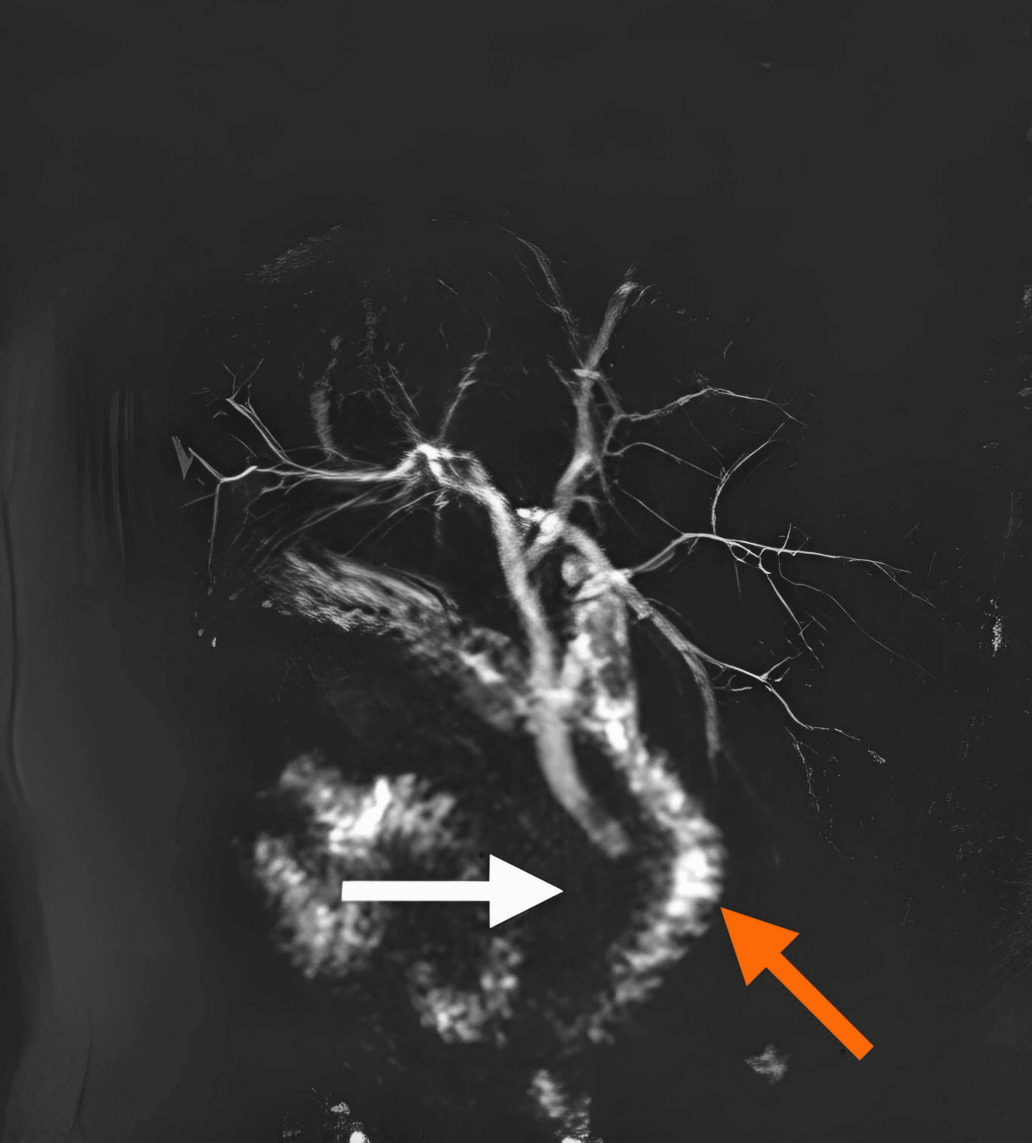
MRCP Void within the gallbladder (white arrow); the duodneum (orange arrow) MRCP: Magnetic resonance cholangiopancreatography

After several months of the previous procedure, the patient visited the clinic to schedule a laparoscopic cholecystectomy. At this time, the abdominal pain was less severe, and his nausea and vomiting had lessened. The patient did not report fever, jaundice, changes in bowel movements, or urinary changes. The patient's vital signs were within the acceptable range, and abdominal examination revealed a normal shape and contour, with mild pain in the left upper quadrant. Baseline investigations, including a complete blood count (CBC), liver function tests (LFTs), kidney function tests (KFTs), and a coagulation profile, were within normal limits. The patient underwent several imaging tests, including an abdominal ultrasound (US) revealing gallstones (Figure 1A) and the spleen positioned on the right side (Figure 1B), a chest x-ray (Figure 2A) and abdominal x-ray (Figure 2B), as well as a magnetic resonance cholangiopancreatography (MRCP) (Figure 3).

**Figure 4:**
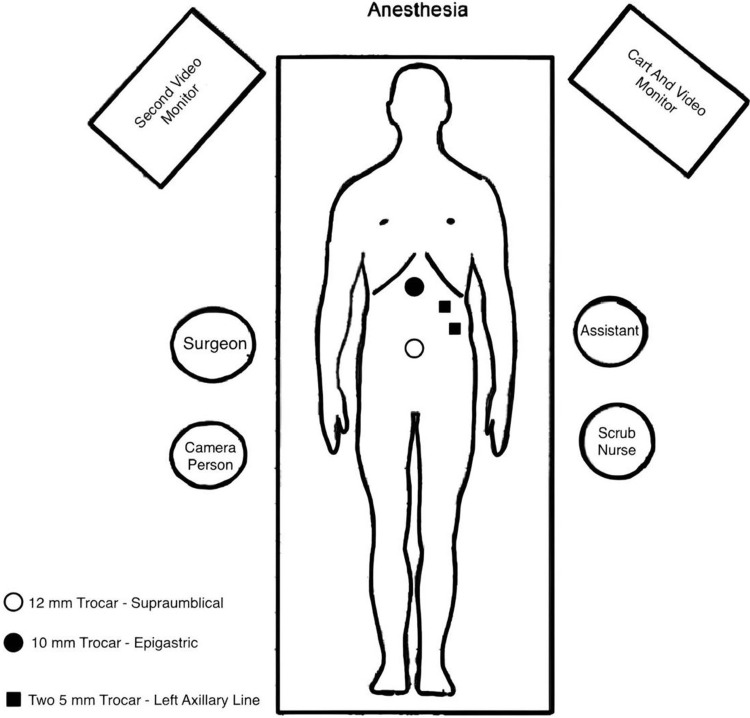
Adapted surgical positioning and trocar site placement for laparoscopic cholecystectomy The surgeon stands facing the left side of the patient's abdomen, while his assistant stands opposed to him in order to accommodate the mirrored anatomical changes

Upon reviewing the patient's lab results and imaging, the general surgery and anesthesiology teams determined that the patient was fit for surgical intervention. The patient was scheduled for a laparoscopic cholecystectomy. During the surgery, the surgeon modified the arrangement of the surgical team and the positioning of the trocar ports to accommodate the procedure (Figure 4). Additionally, the general anesthesia was introduced after positioning the patient supinely.

The abdomen was insufflated using a Veress needle in the right upper quadrant, below the costal margin. Four trocars were inserted under direct vision: a 12 mm trocar at the supraumbilical site, a 12 mm trocar at the epigastric area, and two 5 mm trocars in the left anterior axillary line.

Intraoperative findings revealed a severely adherent gallbladder to the stomach, with the gallbladder impacted in the liver. The adhesions were released using blunt dissection, and the dissection was started at Hartmann's pouch using electrocautery. Calot's triangle was opened, and the cystic duct and artery were skeletonized, clipped, and divided. Sharp and blunt dissection using electrocautery was performed to remove the gallbladder from its bed. The gallbladder was extracted using an endo-bag, and the fascia of the supraumbilical trocar site was closed using a Vicryl 2/0 endo-close assisted device. Hemostasis was achieved, and abdomen was deflated. The trocars were removed, and the skin was cleaned and sutured with a dressing applied.

The patient was successfully extubated and transferred to the general surgery ward for observation. He was discharged home in good condition and followed up in the outpatient department, where he was doing well.

## Discussion

Situs inversus totalis is a uncommon congenital disorder characterized by a generalized defect in the orientation of bodily structures, resulting in a spectrum of laterality disruptions. This condition arises from the inability to establish normal left-right asymmetry, as the cardiac tubes rotate to the left, leading to the heart and other internal organs being positioned in the opposite direction of the normal configuration [6].

Due to the rarity of this condition, healthcare professionals, including gastroenterologists, radiologists, and surgeons, often have limited experience in managing patients with situs inversus [7]. Although the use of CT scanning is the preferable test in patients with situs inversus totalis, several methods of imaging such as MRI, US, CT, and radiography are also effective in identifying the presence of situs inversus [8].

According to a review by Osarenkhoe, cardiac and intestinal diseases have a greater association with situs inversus, and common gastrointestinal comorbidities include gallstones (13.6%), colorectal malignancy (5.8%), and stomach malignancy (5.2%) [9]. In cases of cholelithiasis, most patients present with left-sided abdominal pain, though 10% may experience right-sided abdominal pain, suggesting that the central nervous system may not fully share in the general transposition [2,10].

In 1991, Campos and Sipes executed the very first laparoscopic removal of the gallbladder in a patient with situs inversus totalis, and this remains the standard operation for the treatment of gallstone disease in these patients [4]. However, there is no recognized standard approach, and additional safety measures are proposed to eliminate associated biliary tract anomalies or bile duct injuries [11]. There are several obstacles right-handed surgeons may confront, and they include, but are not limited to, equipment overlapping, resultant early weariness, the necessity to change position in the middle of the operation, and hyperfixation of the surgeon's trunk and left hand [12]. According to a study done by Enciu et al., there was a substantial link between the duration of the surgery and the surgeon's dominant hand, with rapid results being linked with left-handed surgeons. Also, the use of appropriate preoperative imaging techniques, including CT, MRCP, ERCP, and MRI, also helped to reduce the duration of the surgical procedure [13]. As reported by Alkhlaiwy et al., significantly, none of the 91 cases of situs inversus totalis reported in literature after the first successful laparoscopic cholecystectomy in 1991 have reported any complications or converted to open surgery. Furthermore, the complications rate were comparable to those observed in the general population [14]. Also, certain complication as gallstone ileus do not impact the typical presentation even in patients with situs inversus totalis [15]. 

Extensive and careful preoperative assessment along with a good grasp of anatomic knowledge are required in order to ensure a positive results for these patients as there is no universally accepted method.

## Conclusions

Situs inversus is a unique congenital anomaly that poses unique challenges in the management of abdominal pathologies, including gallbladder disorders. Performing laparoscopic cholecystectomy in patients with situs inversus totalis requires exceptional visual-motor skills, extensive reorientation, and thorough preoperative planning to navigate the mirrored anatomy successfully. This case report emphasizes the importance of a multidisciplinary team approach, comprehensive anatomical knowledge, and effective communication among healthcare professionals to ensure the safe and efficient management of patients with this rare condition. Continued research and the sharing of experiences in the medical literature are essential to develop standardized best practices and optimize patient outcomes.
